# Pyrimidine-based twisted donor–acceptor delayed fluorescence molecules: a new universal platform for highly efficient blue electroluminescence†
†Electronic supplementary information (ESI) available: General methods, synthesis and characterization data for intermediates, additional computational and photophysical data, TGA data, and OLED device characteristics. CCDC 1500786. For ESI and crystallographic data in CIF or other electronic format see DOI: 10.1039/c6sc03793c



**DOI:** 10.1039/c6sc03793c

**Published:** 2016-09-26

**Authors:** In Seob Park, Hideaki Komiyama, Takuma Yasuda

**Affiliations:** a INAMORI Frontier Research Center (IFRC) , Kyushu University , 744 Motooka, Nishi-ku , Fukuoka 819-0395 , Japan . Email: yasuda@ifrc.kyushu-u.ac.jp; b Department of Applied Chemistry , Graduate School of Engineering , Kyushu University , 744 Motooka, Nishi-ku , Fukuoka 819-0395 , Japan

## Abstract

Deep-blue emitters that can harvest both singlet and triplet excited states to give high electron-to-photon conversion efficiencies are highly desired for applications in full-color displays and white lighting devices based on organic light-emitting diodes (OLEDs). Thermally activated delayed fluorescence (TADF) molecules based on highly twisted donor–acceptor (D–A) configurations are promising emitting dopants for the construction of efficient deep-blue OLEDs. In this study, a simple and versatile D–A system combining acridan-based donors and pyrimidine-based acceptors has been developed as a new platform for high-efficiency deep-blue TADF emitters. The designed pre-twisted acridan–pyrimidine D–A molecules exhibit small singlet–triplet energy splitting and high photoluminescence quantum yields, functioning as efficient deep-blue TADF emitters. The OLEDs utilizing these TADF emitters display bright blue electroluminescence with external quantum efficiencies of up to 20.4%, maximum current efficiencies of 41.7 cd A^–1^, maximum power efficiencies of 37.2 lm W^–1^, and color coordinates of (0.16, 0.23). The design strategy featuring such acridan–pyrimidine D–A motifs can offer great prospects for further developing high-performance deep-blue TADF emitters and TADF-OLEDs.

## Abstract 2

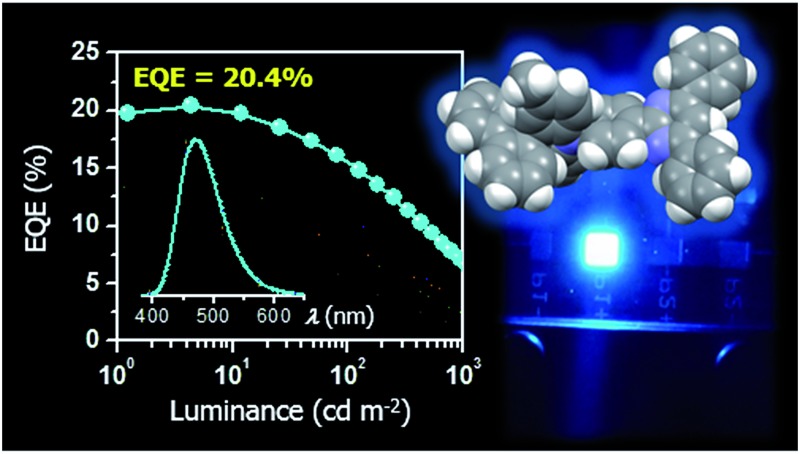
High-efficiency deep blue thermally activated delayed fluorescence (TADF) emitters consisting of acridan–pyrimidine donor–acceptor motifs are developed.

## Introduction

Organic light-emitting diodes (OLEDs) have made great progress towards applications in next-generation flat-panel displays and solid-state lighting over the past three decades since the pioneering work of Tang and VanSlyke in 1987.^1^ To produce full-color displays and white lighting devices based on OLED technologies, the three primary RGB (red, green, and blue) colors are indispensable. Up to date, red and green phosphorescent emitters based on organometallic iridium or platinum complexes primarily match the requirements of application in terms of efficiency, stability, and color purity.^2–5^ However, the overall device performance of blue (especially deep-blue) OLEDs, based on phosphorescent emitters^6^ or conventional fluorescent emitters,^7^ still lags behind its red and green counterparts. Hence, further improvement in the electroluminescence (EL) efficiency, operational stability, and color index is required. Driven by such technological demands, it is vital to develop highly efficient deep-blue emitters with Commission Internationale de l'Éclairage chromaticity coordinate (CIE_*x*,*y*_) values below 0.15, matching closely with the National Television System Committee (NTSC) standard pure blue coordinates of (0.14, 0.08).

Over the last few years, OLEDs utilizing thermally activated delayed fluorescence (TADF) emitters, which can harvest both triplet (T_1_) and singlet (S_1_) excitons for EL *via* efficient reverse intersystem crossing (RISC), have shown conspicuous improvement in device efficiencies, achieving internal EL quantum efficiencies (*η*_int_) of nearly 100%.^8–14^ Replacing phosphorescent organometallic emitters with efficient metal-free pure-organic TADF emitters offers the possibility to not only reduce the cost of materials by eliminating the need for expensive precious metals but also to solve the stability issue of the existing blue phosphorescent materials and their devices. In general, TADF emitters are composed of electron donor (D) and acceptor (A) moieties, which give rise to a small spatial overlap between the highest occupied molecular orbital (HOMO) and the lowest unoccupied molecular orbital (LUMO) in order to minimize the singlet–triplet energy splitting (Δ*E*_ST_) and thereby accelerate the RISC process from its non-radiative T_1_ to radiative S_1_ states. Based on this design principle, various D–A and D–A–D structured blue/sky-blue TADF emitters containing triazine,^9,15–17^ benzosulfone,^10,18–22^ phenone,^23–26^ benzonitrile,^8,27–32^ or phenylborane^33–37^ as the A moiety have recently been synthesized and applied in TADF-OLEDs. However, high-performance blue TADF emitters are still very rare and only a few of them can achieve both a high external EL quantum efficiency (*η*_ext_) exceeding 20% and a suitable color purity with a CIE_*y*_ value below 0.25.^10,16,21,22,29,33,34,38^ Hence, it remains quite challenging to search for an appropriate combination of D and A moieties to simultaneously attain both excellent EL efficiency and high color purity for deep-blue TADF materials.

**Fig. 1:**
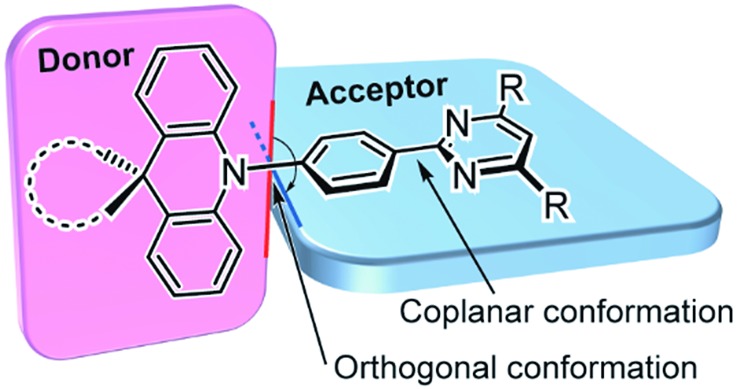
Molecular design and preferred geometry of deep-blue TADF emitters based on pre-twisted acridan–pyrimidine D–A structures.

Herein, we report a new family of highly efficient deep-blue TADF emitters based on a simple pre-twisted D–A architecture (Fig. 1) in which a pyrimidine-based acceptor moiety is connected with a spiroacridan/acridan-based donor moiety through a phenylene π-spacer. Owing to the large steric repulsion between the hydrogen atoms of the acridan unit and the adjacent phenylene spacer, this D–A system offers nearly orthogonal conformations in the ground (S_0_) and S_1_ states, leading to an effective spatial separation of the HOMO and LUMO and a reduction in Δ*E*_ST_. Hence it enables efficient upconversion from the T_1_ to the S_1_ state. We envisage that the pyrimidine unit can serve as a universal building block for deep-blue TADF materials as it possesses a weaker electron-accepting nature than the widely used triazine unit and thus increases the bandgap energy (*E*_g_) and S_1_ and T_1_ energy levels of the resulting D–A molecules. Moreover, the pyrimidine unit can be substituted with a variety of functional groups and fine-tuning of the photophysical and electronic properties can be achieved with simple chemical modifications.

## Results and discussion

### Molecular design and synthesis

**Fig. 2:**
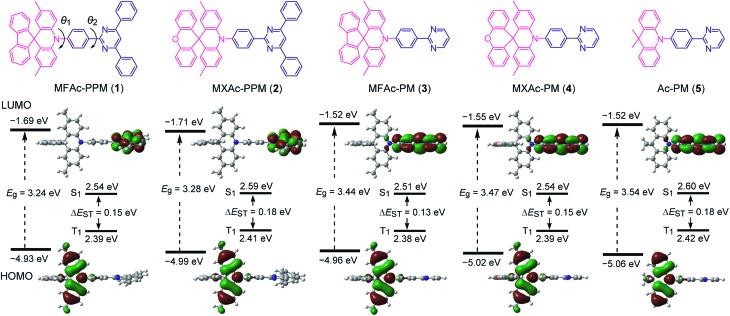
Chemical structures (upper), HOMO and LUMO distributions, and calculated singlet (S_1_) and triplet (T_1_) energy levels (lower) for D–A molecules **1–5** characterized using TD-DFT at the PBE1PBE/6-31G(d) level.

As shown in Fig. 2, we designed a new series of D–A molecules, **1–5** consisting of 2,4,6-triphenylpyrimidine (PPM) or 2-phenylpyrimidine (PM) as an acceptor and spiro[2,7-dimethylacridan-9,9′-fluorene] (MFAc), spiro[2,7-dimethylacridan-9,9′-xanthene] (MXAc), or 9,9-dimethylacridan (Ac) as a donor. The selection of the PPM and PM units which have relatively weak electron-withdrawing characteristics and intrinsic high T_1_ energies is key to producing wide-bandgap deep-blue TADF materials. Our design strategy is justified by time-dependent density functional theory (TD-DFT) calculations, which provide insights into the geometrical and electronic properties of **1–5** at the molecular level. As can be seen from Fig. 2, all of these molecules adopt highly twisted D–A conformations in their optimized geometries, with dihedral angles between the acridan unit and the adjacent phenylene ring (*θ*_1_) of 87–90° owing to the steric repulsion arising from their *peri*-hydrogen atoms. Meanwhile, the dihedral angles between the pyrimidine ring and the central phenylene ring (*θ*_2_) were rather small (<6°). Such nearly orthogonal molecular structures formed by **1–5** can effectively break the π-conjugation between the donor and acceptor moieties and cause localization of the HOMO and LUMO primarily on the acridan and PPM (or PM) units, respectively. Besides, the calculated first excited S_1_ states for **1–5** were dominated by the HOMO → LUMO intramolecular charge-transfer (ICT) transition. As a result, small Δ*E*_ST_ values in the range of 0.13–0.18 eV were estimated for **1–5** from the calculated S_1_ and T_1_ energies (Fig. 2), allowing for efficient RISC and consequently resulting in TADF emission.

**Fig. 3:**
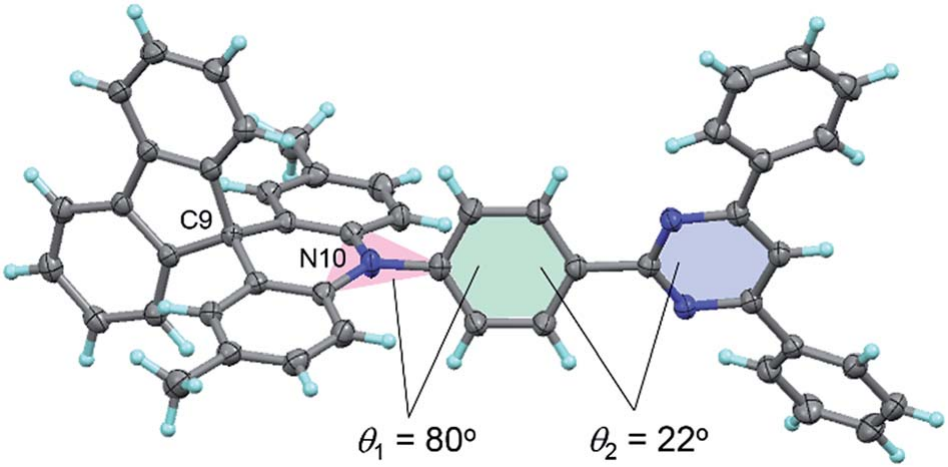
ORTEP diagram of **1** with 50% probability ellipsoids. Atom color code: C, gray; N, blue; H, light-blue.

The configuration of **1** was further verified using X-ray crystallographic analysis (Fig. 3). As per our design, **1** revealed a highly twisted molecular structure with a dihedral angle between the acridan unit and the adjacent phenylene ring of 80°, reasonably consistent with the TD-DFT calculations. It is also noted that the spiro-fused fluorene substituent caused a slight bending of the acridan unit along the C9–N10 axis, on account of the sp^3^ character of the C9 atom.

Compounds **1–5** were readily synthesized in high yields of over 90% through the Buchwald–Hartwig amination of bromo-PPM (for **1** and **2**) or bromo-PM (for **3–5**) with the corresponding spiroacridan/acridan by employing a Pd(OAc)_2_/P(*t*-Bu)_3_HBF_4_ catalytic system. All final products were purified using temperature-gradient vacuum sublimation to obtain highly pure materials for subsequent measurements and device fabrication. The chemical structures of **1–5** were confirmed using ^1^H and ^13^C NMR spectroscopy, mass spectrometry and elemental analysis. The detailed synthetic procedures and characterization data are described in the Experimental section and ESI.† The thermal properties of **1–5** were examined using thermogravimetric analysis (ESI†). Among these new compounds, **1** and **2** possessed the highest thermal stability with a decomposition temperature (*T*_d_, corresponding to 5% weight loss) of 422 °C. Such a *T*_d_ value was much higher than those of **3–5** (*T*_d_ = 351, 354, and 288 °C, respectively). The D–A molecules bearing the spiro-fused D units (MFAC and MXAc) were found to exhibit better thermal properties than that with the non-spiro Ac unit.

### Photophysical and TADF properties

**Fig. 4:**
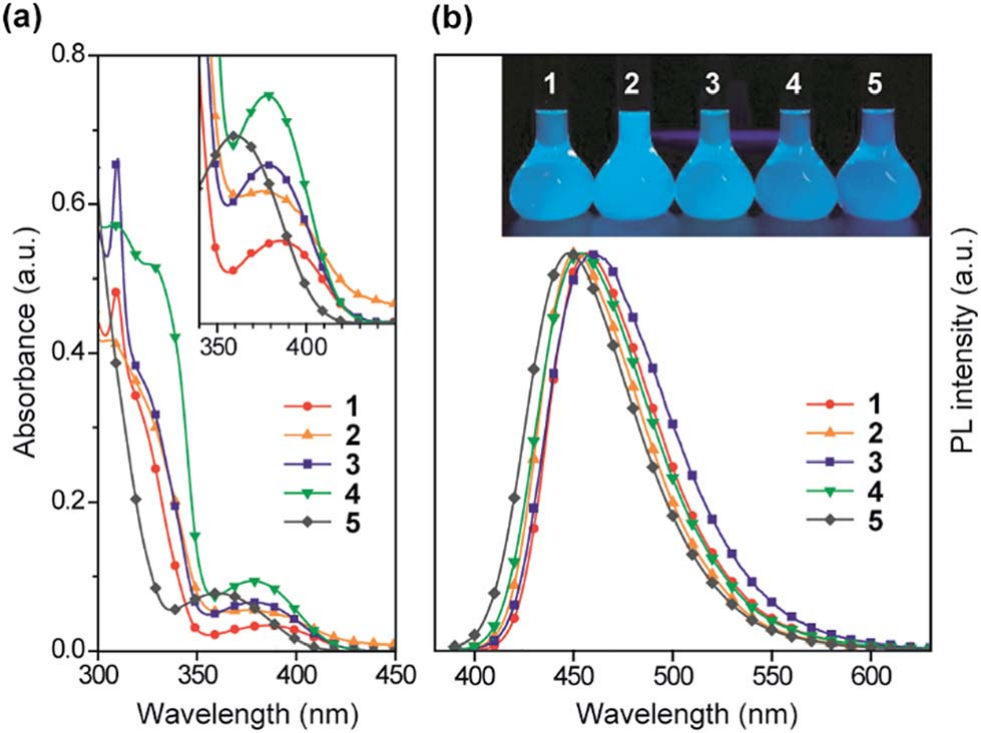
(a) UV-vis absorption and (b) PL spectra of **1–5** in toluene (10^–5^ M). The insets of (a) and (b) represent a magnified view of the lower-energy ICT absorptions and a photograph of the deep-blue PL emission from their solutions under UV irradiation, respectively.

The steady-state UV-vis absorption and photoluminescence (PL) spectra of **1–5** in dilute solution are depicted in Fig. 4 and their relevant photophysical data are summarized in Table 1. All these compounds exhibit similar spectral features which involve two major absorption bands. While the stronger higher-energy absorptions below 330 nm are attributed to the π–π* transitions of the conjugated aromatic units, the much weaker lower-energy absorptions spanning the range of 350–400 nm are assigned to the ICT transitions associated with electron transfer from the acridan to the pyrimidine moieties. Upon photoexcitation at the ICT absorption band, **1–5** in toluene solution exhibited intense deep-blue PL emission with peaks (*λ*_PL_) ranging from 448 to 460 nm.

**Table 1:** Photophysical data for deep-blue TADF emitters **1–5**

	*λ*_abs_ ^*a*^ (nm)	*λ*_PL_ ^*a*^ (nm)	*λ*_PL_ ^*b*^ (nm)	CIE^*b*^ ^,^ ^*c*^ (*x*, *y*)	*Φ*_PL_ ^*b*^ ^,^ ^*d*^ (%)	*τ*_p_ ^*e*^ (ns)	*τ*_d_ ^*e*^ (μs)	HOMO^*f*^ (eV)	LUMO^*g*^ (eV)	*E*_S_ ^*h*^ (eV)	*E*_T_ ^*h*^ (eV)	Δ*E*_ST_ ^*i*^ (eV)
**1**	309, 386	458	464	(0.15, 0.15)	87	12	38	–5.62	–2.67	3.07	2.82	0.25
**2**	306, 378	451	452	(0.15, 0.12)	69	11	40	–5.65	–2.68	3.10	2.85	0.25
**3**	310, 380	461	466	(0.15, 0.18)	91	13	45	–5.60	–2.69	3.06	2.80	0.26
**4**	310, 379	454	458	(0.15, 0.13)	90	11	70	–5.65	–2.70	3.09	2.80	0.29
**5**	286, 359	448	457	(0.15, 0.13)	83	11	78	–5.68	–2.70	3.10	2.80	0.30

^*a*^Measured in toluene solution (10^–5^ M) at room temperature.

^*b*^Measured in 18 wt%-doped thin films in a PPF solid host matrix at room temperature.

^*c*^Commission Internationale de l'Éclairage (CIE) color coordinates.

^*d*^Absolute PL quantum yield evaluated using an integrating sphere under N_2_.

^*e*^PL lifetimes of the prompt (*τ*_p_) and delayed (*τ*_d_) decay components for the 18 wt%-doped films measured at room temperature.

^*f*^Determined using photoelectron yield spectroscopy in neat films.

^*g*^LUMO = HOMO + *E*_g_, in which the optical energy gap (*E*_g_) was derived from the absorption onset of the neat film.

^*h*^Lowest singlet (*E*_S_) and triplet (*E*_T_) energies estimated from the onset wavelengths of the PL spectra at 300 and 5 K in the doped films, respectively.

^*i*^Singlet–triplet energy splitting determined experimentally using Δ*E*_ST_ = *E*_S_ – *E*_T_.

**Fig. 5:**
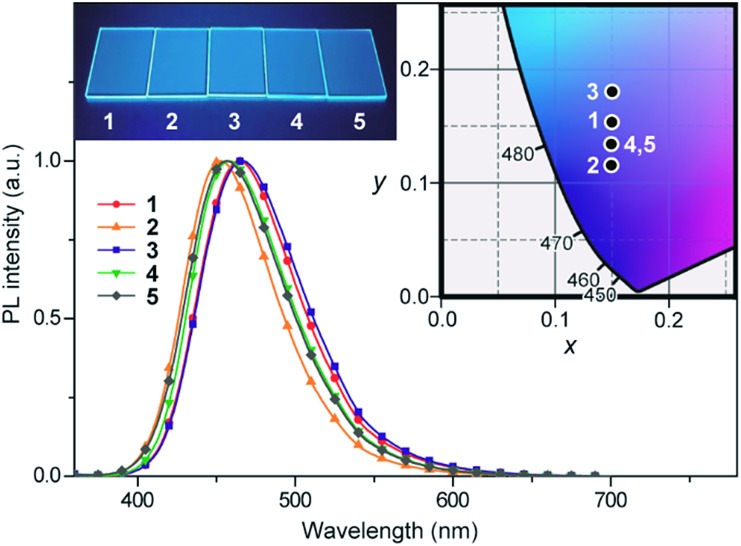
PL spectra of **1–5** in 18 wt%-emitter:PPF doped thin films. The inset shows the CIE chromaticity coordinates and a photograph of the deep-blue PL emission of **1–5** in the doped films.

The photophysical and TADF properties of **1–5** were examined using doped thin films in a solid host matrix. The S_1_ and T_1_ energies (*E*_S_ and *E*_T_, respectively) of **1–5** were determined from the onset of the fluorescence (300 K) and phosphorescence (5 K) spectra, respectively, and thus their Δ*E*_ST_ values were experimentally evaluated to be between 0.25–0.30 eV (Table 1 and ESI†). Because of the high *E*_S_ and *E*_T_ values of 3.0–3.1 eV and 2.8–2.9 eV, respectively, for these wide-bandgap emitters **1–5**, we selected 2,8-bis(diphenylphosphoryl)dibenzo[*b*,*d*]furan (PPF)^39^ with a high *E*_T_ of 3.1 eV as a suitable host material to prevent the reverse energy transfer from the T_1_ states of the guest emitter to the host material and to confine the excitons in the emitters. As shown in Fig. 5, the PL emission from these doped films thoroughly originated from their guest emitters (**1–5**), manifesting an efficient host-to-guest energy transfer. Among these derivatives, MFAc-containing **1** and **3** showed slightly red-shifted PL emissions centered at 464 and 466 nm, respectively, compared with their MXAc-containing counterparts (*λ*_PL_ = 452 and 458 nm for **2** and **4**, respectively), presumably because of the enhanced electron-donating effects caused by the conjugated spirofluorene substituent on the C9 position of the acridan unit. The absolute PL quantum yields (*Φ*_PL_) of the doped films of **1–5** in PPF are as high as 87%, 69%, 91%, 90%, and 83% under N_2_, respectively, which are much higher than those obtained in dilute solutions (*Φ*_PL_ = 32–36% in deoxygenated toluene solution). Such a PL enhancement in the solid state originates from the suppression of the non-radiative deactivation processes caused by collisional and intramolecular rotational excited-energy loss. It is noteworthy that most of these derivatives exhibited CIE_*x*,*y*_ values below 0.15 in those solid thin films, demonstrating their suitability as efficient deep-blue emitters in TADF-OLEDs.

**Fig. 6:**
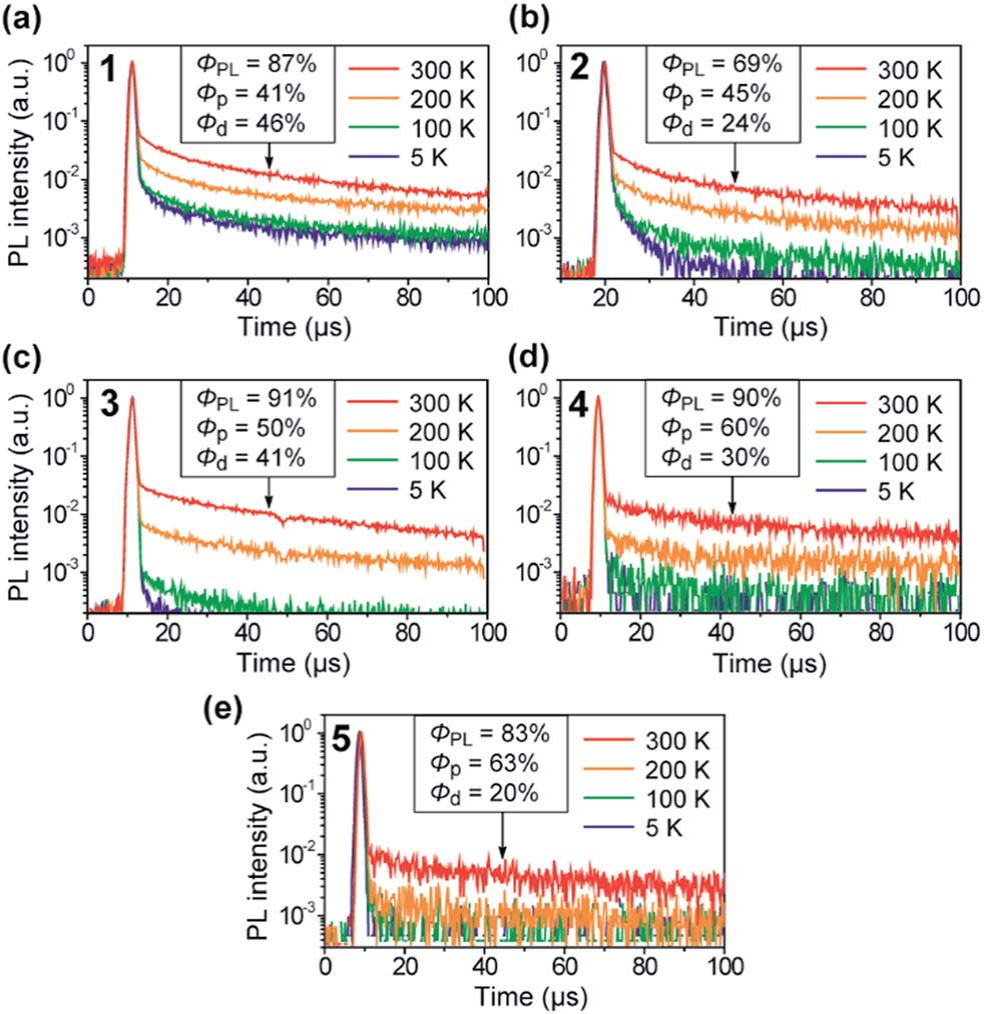
Temperature dependence of the transient PL decay for **1–5** in 18 wt%-emitter:PPF doped thin films in the temperature range of 5–300 K under vacuum.

The TADF characteristics of **1–5** in the doped films were further evidenced by investigating the temperature-dependent transient PL decay. As shown in Fig. 6, each of the transient PL curves displays a clear double-exponential decay profile with prompt and delayed components in oxygen-free conditions. While the prompt component with a lifetime (*τ*_p_) of 11–13 ns corresponds to conventional fluorescence (S_1_ → S_0_), the delayed component with a lifetime (*τ*_d_) of 38–78 μs can be assigned to TADF involving ISC and RISC processes (S_1_ → T_1_ → S_1_ → S_0_). In comparison with **4** and **5**, the relatively shorter *τ*_d_ for **1–3** can be attributed to their smaller Δ*E*_ST_ values (Table 1). Furthermore, the transient PL profiles of the doped films reveal a typical TADF feature:^8^ the PL intensity for the delayed component gradually increases when increasing the temperature from 5 to 300 K. These observations unambiguously indicate that **1–5** can indeed utilize T_1_ excitons for efficient light emission from the S_1_ state *via* the T_1_ → S_1_ thermal upconversion. From the overall *Φ*_PL_ value and the proportion of the integrated areas of the two components in each transient PL curve, the fractional quantum efficiencies for the prompt (*Φ*_p_) and delayed (*Φ*_d_) components were evaluated for the doped films of **1–5**, as given in the insets of Fig. 6. Obviously, these doped films exhibited a high ratio of *Φ*_d_ with respect to the overall *Φ*_PL_ at ambient temperature (300 K), suggesting that a large portion of the S_1_ excitons underwent efficient ISC and RISC and then decayed to emit delayed fluorescence upon photoexcitation. Indeed, for **1–5**, high RISC efficiencies (*Φ*_RISC_) of 44–82% were assumed by the equation:^22^ *Φ*_RISC_ = *Φ*_d_/(1 – *Φ*_p_) (see the ESI for details†).

### Electroluminescence performance

**Fig. 7:**
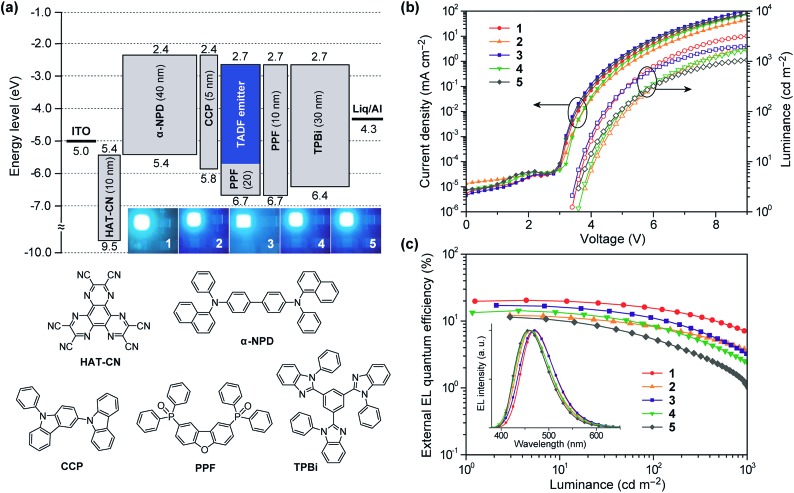
(a) Schematic energy-level diagram and photos of the EL emission for the blue TADF-OLEDs based on **1–5** as emitters (top) and chemical structures of the materials used in the devices (bottom). (b) Current density and luminance *versus* voltage (*J*–*V*–*L*) characteristics and (c) external EL quantum efficiency *versus* luminance (*η*_ext_–*L*) characteristics of the blue TADF-OLEDs. The inset of (c) represents the EL spectra measured at 10 mA cm^–2^.

To investigate the EL performance of deep-blue TADF emitters **1–5**, multilayer OLEDs were fabricated by employing thin films of **1–5** doped in a PPF host as an emitting layer (EML). We adopted the following device configuration: indium-tin-oxide (ITO, 100 nm)/HAT-CN (10 nm)/α-NPD (40 nm)/CCP (5 nm)/EML (20 nm)/PPF (10 nm)/TPBi (30 nm)/Liq (1 nm)/Al (100 nm), as illustrated in Fig. 7a. In this device architecture, HAT-CN (2,3,6,7,10,11-hexacyano-1,4,5,8,9,12-hexaazatriphenylene) and α-NPD (4,4′-bis-[*N*-(1-naphthyl)-*N*-phenylamino]-1,1′-biphenyl) were used as a hole-injection layer and a hole-transporting layer, respectively; whereas, TPBi (1,3,5-tris(*N*-phenylbenzimidazol-2-yl)benzene) and Liq (8-hydroxyquinoline lithium) served as an electron-transporting layer and an electron-injection material, respectively. Additionally, thin layers of CCP^33^ (9-phenyl-3,9′-bicarbazole) and PPF^39^ with a high *E*_T_ of 3.0 and 3.1 eV were inserted as exciton-blocking layers to suppress the triplet exciton deactivation at the neighboring interfaces and to confine the excitons within the EML.

The EL characteristics of the fabricated TADF-OLEDs are depicted in Fig. 7b and c, and the key device parameters are compiled in Table 2. The devices based on **1–5** displayed bright blue EL emission peaking in the range of 458–470 nm, with rather low turn-on voltages (*V*_on_) of 3.4–3.6 V. Their EL spectra were consistent with the corresponding PL spectra, suggesting efficient carrier injection, transport, and recombination into the EML within the device. Among the fabricated devices, the device employing **1** achieved the highest EL efficiencies with a maximum *η*_ext_ of 20.4%, current efficiency (*η*_c_) of 41.7 cd A^–1^, and power efficiency (*η*_p_) of 37.2 lm W^–1^ at low current densities without any light out-coupling enhancement. The CIE coordinates of the EL from this device were (0.16, 0.23). To our knowledge, these efficiencies are among the highest level for blue TADF-OLEDs ever reported.^10,16,21,22,29,33,34,38^ So far, deep-blue TADF-OLEDs with emission maxima (*λ*_EL_) below 470 nm have rarely achieved a high *η*_ext_ exceeding 20%. Moreover, the device employing **1** also showed a relatively reduced efficiency roll-off compared to the other devices; the *η*_ext_ value still remained as high as 15.6% at a practical luminance of 100 cd m^–2^. The reduced roll-off behavior for **1** can be attributed to the fast RISC originating from its relatively shorter *τ*_d_ and the suppression of triplet–triplet annihilation (TTA) and singlet–triplet annihilation (STA),^40,41^ as discussed below.

**Table 2:** EL performance of the TADF-OLEDs based on **1–5**

Emitter^*a*^	**1**	**2**	**3**	**4**	**5**
*λ*_EL_ ^*b*^ (nm)	470	462	469	460	458
*V*_on_ ^*c*^ (V)	3.4	3.6	3.4	3.6	3.6
*η*_ext,max_ ^*d*^ (%)	20.4	12.2	17.1	14.3	11.4
*η*_ext,100_ ^*e*^ (%)	15.6	8.2	10.9	8.4	5.4
*η*_c_ ^*f*^ (cd A^–1^)	41.7	22.7	34.3	25.0	18.9
*η*_p_ ^*g*^ (lm W^–1^)	37.2	18.8	31.7	20.7	16.5
CIE^*h*^ (*x*, *y*)	(0.16, 0.23)	(0.16, 0.20)	(0.16, 0.21)	(0.16, 0.19)	(0.15, 0.15)

^*a*^Device configuration: ITO/HAT-CN (10 nm)/α-NPD (40 nm)/CCP (5 nm)/18 wt%-emitter:PPF (20 nm)/PPF (10 nm)/TPBi (30 nm)/Liq (1 nm)/Al (100 nm).

^*b*^EL emission maximum.

^*c*^Turn-on voltage at a brightness of 1 cd m^–2^.

^*d*^Maximum external EL quantum efficiency.

^*e*^External EL quantum efficiency at 100 cd m^–2^.

^*f*^Maximum current efficiency.

^*g*^Maximum power efficiency.

^*h*^Commission Internationale de l'Éclairage (CIE) chromaticity coordinates recorded at 10 mA cm^–2^.

Comparing the performance of the TADF-OLEDs containing **1–5**, the maximum *η*_ext_ values were in the order of **1** (20.4%) > **3** (17.1%) > **4** (14.3%) > **2** (12.2%) > **5** (11.4%). The relatively lower efficiencies of the devices with **2** and **5** compared to those with **1**, **3**, and **4** can be mainly ascribed to their lower *Φ*_PL_ and *Φ*_d_ values. Nevertheless, the *η*_ext_ values of **2** and **5** were more than two times higher than those expected from conventional fluorescent emitters with the same *Φ*_PL_ values. These pyrimidine-based deep-blue TADF emitters could thus achieve high EL efficiencies by utilizing both the electro-generated T_1_ and S_1_ excitons for efficient light emission. However, the EL efficiencies for some of these TADF-OLEDs significantly decreased with increasing current density (or luminance). This severe efficiency roll-off is primarily attributed to the long-lived excited states of the T_1_ excitons, which undergo exciton deactivation processes such as TTA and STA. The TTA model is used here to analyze the efficiency roll-off for the devices containing **1–5**, according to the following equation:^40–42^ 
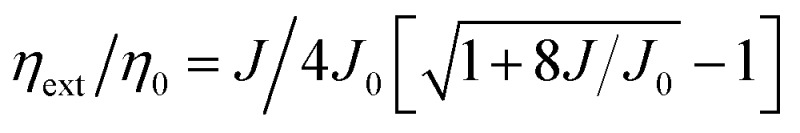
where *η*_0_ is the external EL quantum efficiency in the absence of TTA and *J*_0_ is the critical current density at *η*_ext_ = *η*_0_/2. The fitted curves based on the TTA model agreed well with the experimental *η*_ext_–*J* plots for all the devices containing **1–5** with correlation coefficients greater than 0.98 (ESI†), which indicates that the efficiency roll-off for these devices was primarily caused by TTA exciton deactivation. Indeed, the device based on **5** showed a smaller *J*_0_ value (0.9 mA cm^–2^) than that of **1** (2.1 mA cm^–2^), which implies that **5** suffered from more severe TTA and efficiency roll-off as the current density increased. This propensity arises from the relatively long TADF lifetime (*τ*_d_) of **5** in the doped film. If efficient deep-blue TADF emitters with a much shorter *τ*_d_ (<1 μs) can be realized, we can therefore expect that high *η*_ext_ values of over 20% can be retained even at higher current densities.

## Conclusions

A new family of deep-blue TADF emitters, consisting of pre-twisted acridan–pyrimidine D–A motifs, were designed and synthesized. All of these emitters in doped thin films showed excellent PL properties with quantum yields of 69–91% accompanied by prominent TADF originating from their small Δ*E*_ST_. By employing these TADF emitters for OLEDs, considerably high maximum external EL quantum efficiencies of up to 20.4% with CIE coordinates of (0.16, 0.23) were achieved. Deep-blue EL with CIE coordinates of (0.15, 0.15) could also be obtained through rational molecular design in this platform. These results validate a versatile design strategy to utilize pyrimidine derivatives as a universal platform for the further development of efficient deep-blue organic emitters.

## Experimental section

### Materials and synthesis

**Scheme 1:**
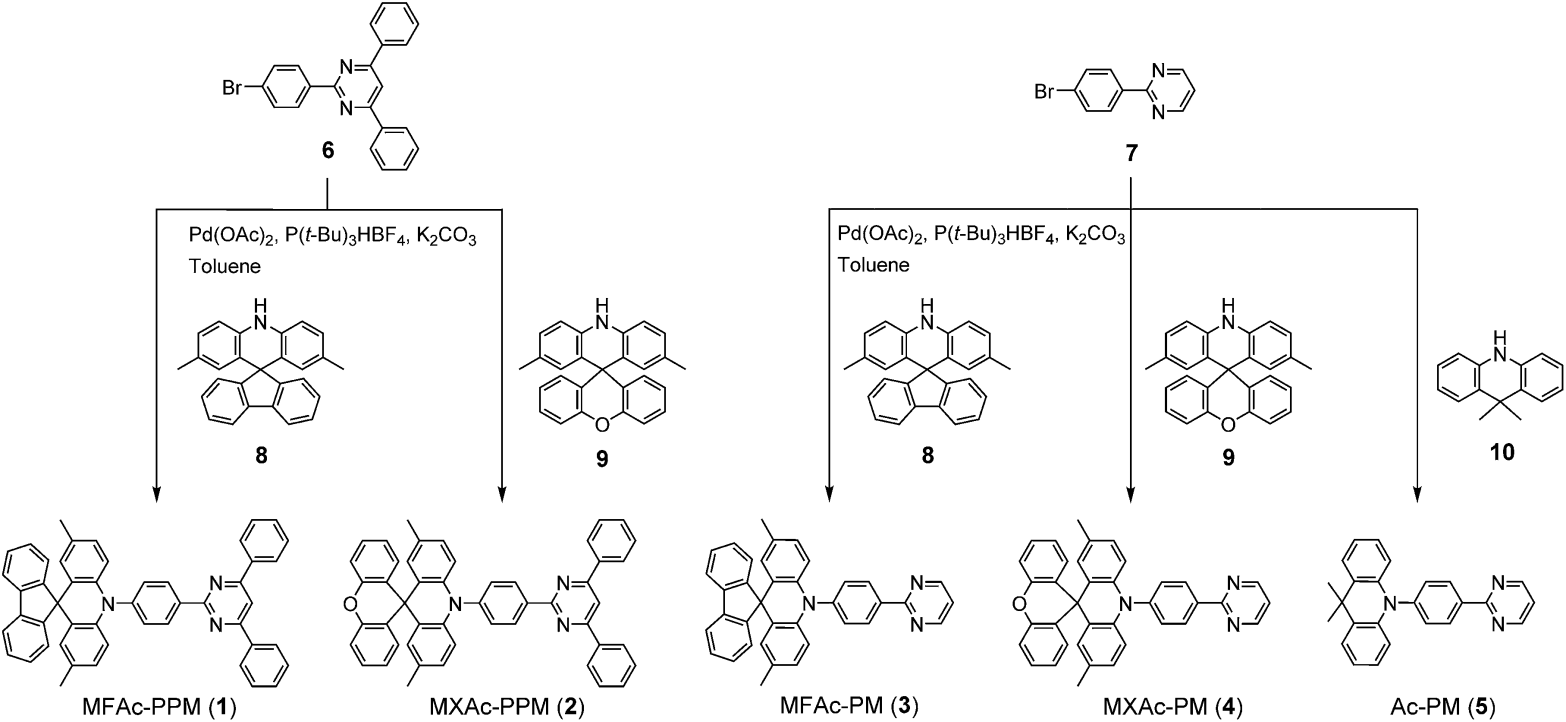
Synthesis routes for pyrimidine-based TADF molecules **1–5**.

All commercially available reagents and solvents were used as received unless otherwise noted. 2,8-Bis(diphenylphosphoryl)dibenzo[*b*,*d*]furan (PPF)^39^ and 9-phenyl-3,9′-bicarbazole (CCP)^33^ were prepared according to the literature procedures, and were purified using vacuum sublimation. 2,3,6,7,10,11-Hexacyano-1,4,5,8,9,12-hexaazatriphenylene (HAT-CN) was donated by Nippon Soda Co., Ltd. and was purified using vacuum sublimation before use. Other OLED materials were purchased from E-Ray Optoelectronics Technology Co., Ltd. and were used for the device fabrication without further purification. The synthesis routes for deep-blue TADF molecules **1–5** are outlined in Scheme 1, and detailed synthetic procedures and characterization data for other intermediates (**6–9**) are given in the ESI.† 9,9-Dimethylacridan^43^ (**10**) was prepared according to the literature procedure. All final products were purified using temperature-gradient vacuum sublimation with a P-100 system (ALS Technology), before the measurements and device fabrication.

#### MFAc-PPM (**1**)

A mixture of **6** (1.00 g, 2.58 mmol), **8** (0.93 g, 2.59 mmol), Pd(OAc)_2_ (0.022 g, 0.1 mmol), P(*t*-Bu)_3_HBF_4_ (0.03 g, 0.1 mmol), and K_2_CO_3_ (1.07 g, 7.7 mmol) in dry toluene (30 mL) was refluxed for 48 h under N_2_. After cooling to room temperature, the reaction mixture was filtered through a Celite pad and then the filtrate was concentrated under reduced pressure. The product was purified using column chromatography on silica gel (eluent: hexane/chloroform = 3 : 1, v/v) to afford **1** as a white solid (yield = 1.60 g, 93%). ^1^H NMR (400 MHz, DMSO-*d*_6_): *δ* 9.03 (d, *J* = 8.4 Hz, 2H), 8.67 (s, 1H), 8.60–8.58 (m, 4H), 7.99 (d, *J* = 7.6 Hz, 2H), 7.78 (d, *J* = 8.4 Hz, 2H), 7.68–7.66 (m, 6H), 7.45 (td, *J* = 7.4 Hz, 1.3 Hz, 2H), 7.41 (d, *J* = 7.2 Hz, 2H), 7.33 (td, *J* = 7.2 Hz, 1.2 Hz, 2H), 6.80 (dd, *J* = 8.6 Hz, 1.8 Hz, 2H), 6.32 (d, *J* = 8.4 Hz, 2H), 6.04 (d, *J* = 1.6 Hz, 2H), 1.90 (s, 6H). ^13^C NMR (100 MHz, CDCl_3_): *δ* 165.04, 164.02, 156.52, 143.81, 139.24, 139.22, 138.16, 137.39, 131.38, 131.19, 130.98, 129.48, 129.03, 128.40, 128.00, 127.87, 127.45, 127.34, 125.86, 124.63, 119.83, 114.52, 110.55, 56.92, 20.40. MS (MALDI-TOF): *m*/*z* calcd 665.28 [M]^+^; found 665.18. Anal. calcd (%) for C_49_H_35_N_3_: C 88.39, H 5.30, N 6.31; found: C 88.35, H 5.23, N 6.34.

#### MXAc-PPM (**2**)

This compound was synthesized according to the same procedure as described above for the synthesis of **1**, except that **9** (0.97 g, 2.58 mmol) was used as the reactant instead of **8**, yielding **2** as a white solid (yield = 1.60 g, 91%). ^1^H NMR (400 MHz, DMSO-*d*_6_): *δ* 9.01 (d, *J* = 8.8 Hz, 2H), 8.66 (s, 1H), 8.60–8.57 (m, 4H), 7.73 (d, *J* = 8.8 Hz, 2H), 7.67–7.66 (m, 6H), 7.26 (dd, *J* = 6.0 Hz, 1.6 Hz, 4H), 7.15 (dd, *J* = 8.8 Hz, 1.2 Hz, 2H), 7.08–7.04 (m, 2H), 6.76 (dd, *J* = 8.6 Hz, 1.4 Hz, 2H), 6.48 (d, *J* = 2.0 Hz, 2H), 6.24 (d, *J* = 8.4 Hz, 2H), 1.96 (s, 6H). ^13^C NMR (100 MHz, CDCl_3_): *δ* 165.06, 163.99, 148.44, 138.22, 137.38, 137.14, 132.54, 132.06, 131.54, 131.43, 131.25, 130.99, 129.74, 129.52, 129.04, 127.83, 127.45, 127.34, 123.69, 115.85, 114.06, 110.58, 44.74, 20.48. MS (MALDI-TOF): *m*/*z* calcd 681.28 [M]^+^; found 681.11. Anal. calcd (%) for C_49_H_35_N_3_O: C 86.32, H 5.17, N 6.16; found: C 86.45, H 5.11, N 6.31.

#### MFAc-PM (**3**)

A mixture of **7** (1.00 g, 4.25 mmol), **8** (1.55 g, 4.31 mmol), Pd(OAc)_2_ (0.03 g, 0.14 mmol), P(*t*-Bu)_3_HBF_4_ (0.03 g, 0.1 mmol), and K_2_CO_3_ (1.80 g, 13.0 mmol) in dry toluene (80 mL) was refluxed for 12 h under N_2_. After cooling to room temperature, the reaction mixture was filtered through a Celite pad and then the filtrate was concentrated under reduced pressure. The product was purified using column chromatography on silica gel (eluent: hexane/chloroform = 3 : 1, v/v) to afford **3** as a white solid (yield = 2.01 g, 92%). ^1^H NMR (400 MHz, DMSO-*d*_6_): *δ* 9.02 (d, *J* = 4.8 Hz, 2H), 8.77 (dd, *J* = 6.4 Hz, 2.0 Hz, 2H), 7.98 (d, *J* = 7.6 Hz, 2H), 7.71 (dd, *J* = 6.8 Hz, 2.0 Hz, 2H), 7.55 (t, *J* = 4.8 Hz, 1H), 7.44 (td, *J* = 7.2 Hz, 1.2 Hz, 2H), 7.39 (d, *J* = 7.2 Hz, 2H), 7.32 (td, *J* = 7.2 Hz, 1.2 Hz, 2H), 6.78 (dd, *J* = 8.8 Hz, 1.9 Hz, 2H), 6.26 (d, *J* = 8.4 Hz, 2H), 6.03 (d, *J* = 2.0 Hz, 2H), 1.89 (s, 6H). ^13^C NMR (100 MHz, CDCl_3_): *δ* 164.24, 157.44, 156.51, 144.01, 139.24, 139.14, 137.55, 131.58, 130.86, 129.52, 128.39, 127.98, 127.89, 127.45, 125.84, 124.61, 119.82, 119.39, 114.45, 56.88, 20.39. MS (MALDI-TOF) *m*/*z*: calcd 513.22 [M]^+^; found 514.04. Anal. calcd (%) for C_37_H_27_N_3_: C 86.52, H 5.30, N 8.18; found: C 86.64, H 5.06, N 8.23.

#### MXAc-PM (**4**)

This compound was synthesized according to the same procedure as described above for the synthesis of **3**, except that **9** (1.60 g, 4.26 mmol) was used as the reactant instead of **8**, yielding **4** as a white solid (yield = 2.03 g, 90%). ^1^H NMR (400 MHz, DMSO-*d*_6_): *δ* 9.01 (d, *J* = 5.2 Hz, 2H), 8.75 (dd, *J* = 6.4 Hz, 2.0 Hz, 2H), 7.67 (d, *J* = 8.4 Hz, 2H), 7.55 (t, *J* = 5.0 Hz, 1H), 7.25–7.23 (m, 4H), 7.12 (dd, *J* = 7.6 Hz, 1.2 Hz, 2H), 7.06–7.04 (m, 2H), 6.74 (dd, *J* = 8.8 Hz, 1.9 Hz, 2H), 6.46 (d, *J* = 1.6 Hz, 2H), 6.18 (d, *J* = 8.8 Hz, 2H), 1.95 (s, 6H). ^13^C NMR (100 MHz, CDCl_3_): *δ* 164.18, 157.44, 148.41, 143.91, 137.60, 137.04, 132.55, 132.03, 131.62, 131.51, 130.91, 129.77, 129.50, 127.79, 127.43, 123.66, 119.40, 115.83, 113.98, 44.70, 20.46. MS (MALDI-TOF): *m*/*z* calcd 529.22 [M]^+^; found 529.12. Anal. calcd (%) for C_37_H_27_N_3_O: C 83.91, H 5.14, N 7.93; found: C 83.84, H 5.03, N 8.02.

#### Ac-PM (**5**)

This compound was synthesized according to the same procedure as described above for the synthesis of **3**, except that **10** (0.89 g, 4.25 mmol) was used as the reactant instead of **8**, yielding **5** as a white solid (yield = 1.45 g, 94%). ^1^H NMR (400 MHz, DMSO-*d*_6_): *δ* 8.99 (d, *J* = 5.2 Hz, 2H), 8.69 (d, *J* = 8.4 Hz, 2H), 7.55–7.50 (m, 5H), 6.99 (td, *J* = 7.7 Hz, 1.3 Hz, 2H), 6.93 (td, *J* = 7.5 Hz, 1.3 Hz, 2H), 6.24 (dd, *J* = 8.0 Hz, 1.2 Hz, 2H), 1.64 (s, 6H). ^13^C NMR (100 MHz, CDCl_3_): *δ* 164.19, 157.40, 143.66, 140.69, 137.49, 131.55, 130.74, 130.08, 126.38, 125.25, 120.66, 119.35, 114.12, 36.00, 31.28. MS (MALDI-TOF): *m*/*z* calcd 363.17 [M]^+^; found 362.99. Anal. calcd (%) for C_25_H_21_N_3_: C 82.61, H 5.82, N 11.56; found: C 82.61, H 5.75, N 11.65.

### Photophysical measurements

The UV-vis absorption and photoluminescence (PL) spectra were measured with a V-670 spectrometer (Jasco) and a FP-8600 spectrophotometer (Jasco), respectively, using degassed spectral grade solvents. The absolute PL quantum yields (*Φ*_PL_) were determined using an ILF-835 integrating sphere system (Jasco). The transient PL decay measurements were carried out using a C11367 Quantaurus-tau fluorescence lifetime spectrometer (Hamamatsu Photonics; *λ* = 340 nm, pulse width = 100 ps, and repetition rate = 20 Hz) under N_2_, and a C9300 streak camera (Hamamatsu Photonics) with an N_2_ gas laser (*λ* = 337 nm, pulse width = 500 ps, and repetition rate = 20 Hz) under vacuum (<4 × 10^–1^ Pa). The HOMO energies of materials in neat films were determined using an AC-2 ultraviolet photoelectron spectrometer (Riken-Keiki). The LUMO energies were estimated by subtracting the optical energy gaps (*E*_g_) from the measured HOMO energies; the *E*_g_ values were determined from the onset positions of the PL spectra of the thin films.

### OLED fabrication and characterization

ITO-coated glass substrates were cleaned with detergent, deionized water, acetone, and isopropanol. The substrates were then subjected to UV–ozone treatment for 30 min before they were loaded into an E-200 vacuum evaporation system (ALS Technology). The organic layers and a cathode aluminum layer were thermally evaporated onto the substrates under vacuum (<6 × 10^–5^ Pa) with an evaporation rate of <0.3 nm s^–1^ through a shadow mask. The layer thickness and deposition rate were monitored *in situ* during deposition using an oscillating quartz thickness monitor. OLED characteristics were measured using a 2400 source meter (Keithley) and a CS-2000 spectroradiometer (Konica Minolta).
