# Supplementary material for: Pyrimidine-based twisted donor–acceptor delayed fluorescence molecules: a new universal platform for highly efficient blue electroluminescence
Source: Chem Sci. 2016 Sep 26;8(2):953–60. doi: 10.1039/c6sc03793c (PMC5354065; doi:10.1039/c6sc03793c)
Supplement: Supplementary file 1 [file SC-008-C6SC03793C-s001.pdf]

Electronic Supplementary Material (ESI) for Chemical Science.
This journal is © The Royal Society of Chemistry 2016

Electronic Supplementary Information (ESI)

Pyrimidine-based twisted donor–acceptor delayed fluorescence molecules: a new universal platform for highly efficient blue electroluminescence

In Seob Park,[ab] Hideaki Komiyama[a] and Takuma Yasuda*[ab]

[a] INAMORI Frontier Research Center (IFRC), Kyushu University, 744 Motooka, Nishi-ku, Fukuoka 819-0395, Japan

[b] Department of Applied Chemistry, Graduate School of Engineering, Kyushu University, 744 Motooka, Nishi-ku, Fukuoka 819-0395, Japan

[*] E-mail: yasuda@ifrc.kyushu-u.ac.jp

Table of Contents:

General methods. S2
Synthesis and characterization. S2–S5
Table S1 TD-DFT calculation results S5
Determination of rate constants. S6
Table S2 Rate constants and quantum efficiencies of **1–5** S7
Fig. S1 TGA curves of **1–5** S7
Fig. S2 Fluorescence and phosphorescence PL spectra of **1–5** S8
Fig. S3 OLED characteristics of **4** with different doping concentration S8
Fig. S4 Power efficiencies and current efficiencies of TADF-OLEDs S9
Fig. S5 External EL quantum efficiencies fitted by the TTA model S9

General Methods.

^{1}H and ^{13}C NMR spectra were recorded on a Bruker Avance III 400 spectrometer. Chemical shifts of ^{1}H and ^{13}C NMR signals were quoted to tetramethylsilane (δ = 0.00), $CDCl_3$ (δ = 77.0), and DMSO-d_6 (δ = 39.5) as internal standards. Matrix-assisted laser desorption ionization time-of-flight (MALDI-TOF) mass spectra were collected on an Autoflex III spectrometer (Bruker Daltonics) using dithranol as the matrix. Elemental analysis was performed using an MT-5 CHN corder (Yanaco). Single-crystal X-ray analysis was carried out using a Rigaku VariMax with Saturn 70 system with graphite monochromated Mo$K\alpha$ radiation.

Quantum chemical calculations were performed using the Gaussian 09 program package. The molecular geometries in the ground state were optimized using the PBE1PBE functional with the 6-31G(d) basis set in the gas phase. The lowest singlet and triplet excited states were computed using the optimized structures with time-dependent density functional theory (TD-DFT) at the same level.

Synthesis and characterization.

The synthetic routes for intermediates **6–9** are outlined in Schemes S1–S4, respectively. The detailed synthetic procedures and characterization data for intermediates **6–9** are described below.

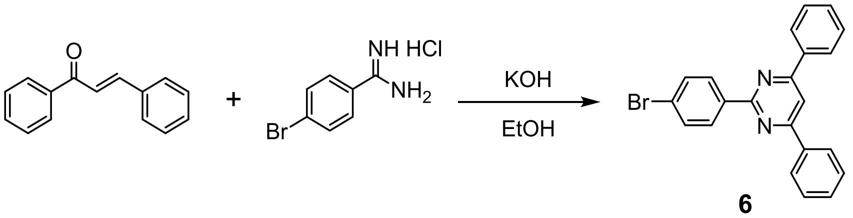

6

Scheme S1. Synthesis of 2-(4-bromophenyl)-4,6-diphenylpyrimidine (**6**).

Compound 6: To a stirred mixture of chalcone (8.84 g, 42.5 mmol) and 4-bromobenzamidine hydrochloride (5.00 g, 21.2 mmol) in ethanol (200 mL) was added dropwise a solution of potassium hydroxide (2.38 g, 42.4 mmol) in ethanol (100 mL) at room temperature. The mixture was then refluxed for 3 h. After cooling to room temperature, the formed precipitate was collected by filtration, washed with a large amount of water, and dried under vacuum to afford **6** as a white solid (yield = 16.0 g, 97%). ^{1}H NMR (400 MHz, $CDCl_3$): δ 8.61 (dd, J = 6.8 Hz, 2.0 Hz, 2H), 8.29-8.26 (m, 4H), 8.04 (s, 1H), 7.66 (dd, J = 6.8 Hz, 2.0 Hz, 2H), 7.59-7.55 (m, 6H). MS (MALDI-TOF): m/z calcd 386.04 $[M]^{+}$; found 386.68.

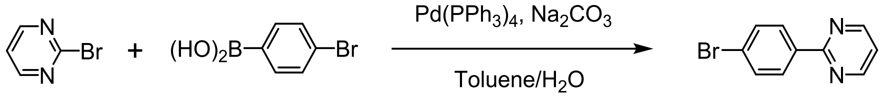

7

Scheme S2. Synthesis of 2-(4-bromophenyl)pyrimidine (**7**).

Compound 7: 2-Bromopyrimidine (10.0 g, 62.9 mmol) and $Pd(PPh_3)_4$ (0.73 g, 0.63 mmol) were dissolved in toluene (100 mL) under N_2 at room temperature. 4-Bromophenylboronic acid (12.6 g, 62.7 mmol) and an aqueous solution (50 mL) of sodium carbonate (20.0 g, 189 mmol) were then added to the solution. The mixture was stirred for 24 h at 90 °C under N_2. After cooling to room temperature, the reaction mixture was added in to water, and the product was extracted with chloroform. The combined organic layers were washed with water and dried over anhydrous Na_2SO_4. After filtration and evaporation, the product was purified by column chromatography on silica gel (eluent: hexane/ethyl acetate = 20:1, v/v) to yield **7** as a white solid (yield = 4.4 g, 30%). ^{1}H NMR (400 MHz, $CDCl_3$): δ 8.74 (d, J = 4.8 Hz, 2H), 8.27 (d, J = 9.0 Hz, 2H), 7.56 (d, J = 9.0 Hz, 2H), 7.15 (t, J = 4.8 Hz, 1H).

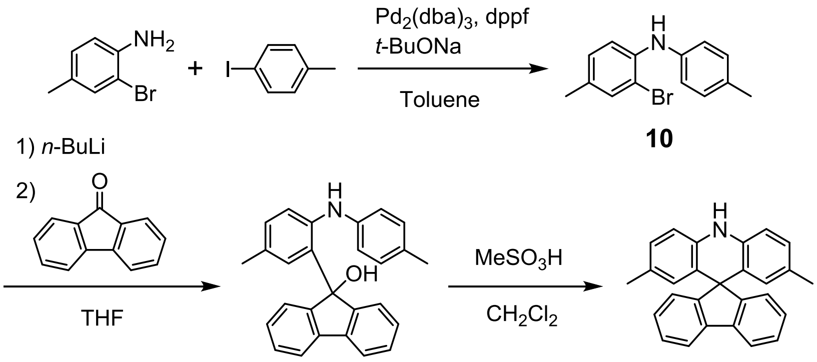

8

Scheme S3. Synthesis of spiro[2,7-dimethylacridine-9,9'-fluorene] (**8**).

Compound 10: To a stirred solution of 2-bromo-4-methylaniline (48.4 g, 260 mmol), sodium *tert*-butoxide (37.5 g, 390 mmol) in dry toluene (520 mL) were added 1-iodo-4-methylbenzene (64.2 g, 286 mmol), $Pd(OAc)_2$ (0.58 g, 2.6 mmol), and 1,1'-bis(diphenylphosphino)ferrocene (dppf, 1.44 g, 2.6 mmol) at room temperature. The mixture was refluxed for 8 h under N_2. After cooling to room temperature, the reaction mixture was filtered through a Celite pad with chloroform, and the filtrate was concentrated under reduced pressure. The crude product was purified by column chromatography on silica gel (eluent: hexane/dichloromethane = 9:1, v/v) to give **10** as a

white solid (yield = 54.8 g, 74%). ^{1}H NMR (400 MHz, $CDCl_3$): δ 7.56 (d, J = 8.3 Hz, 1H), 7.34 (d, J = 1.4 Hz, 1H), 7.10 (d, J = 8.0 Hz, 2H), 7.07 (d, J = 8.3 Hz, 1H), 7.00 (d, J = 8.4 Hz 2H), 6.96-6.91 (m, 1H), 2.32 (s, 3H), 2.25 (s, 3H). MS (MALDI-TOF): m/z calcd 275.03 $[M]^+$; found 275.71.

Compound 8: To a stirred solution of **10** (21.0 g, 76.0 mmol) in dry THF (300 mL) was added dropwise *n*-butyllithium (1.6 M, 97.4 mL, 156 mmol) at −78 °C under N_2. The mixture was stirred for 1 h at that temperature. After heating up to 0 °C, fluorenone (15.1 g, 83.6 mmol) was added to the solution, and the mixture was further stirred for 6 h at room temperature. The reaction was quenched by addition of a large amount of water. Then, the product was extracted with chloroform and dried over anhydrous Na_2SO_4. After filtration and evaporation, the product was used in the next reaction without further purification.

This crude product was dissolved in dichloromethane (300 mL), and methanesulfonic acid (14.6 g, 152 mmol) was added to the solution at room temperature. The mixture was refluxed for 1 h under air. After cooling to room temperature, the mixture was concentrated under reduced pressure. The residue was purified by column chromatography on silica gel (eluent: hexane/dichloromethane = 4:1, v/v) to afford **8** as a white solid (yield = 12.2 g, 45%). ^{1}H NMR (400 MHz, $CDCl_3$): δ 7.78 (br, 1H), 7.37-7.18 (m, 14H). MS (MALDI-TOF): m/z calcd 359.17 $[M]^+$; found, 358.98.

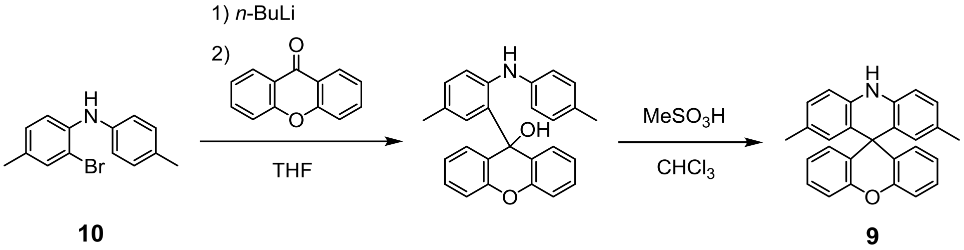

Scheme S4. Synthesis of spiro[2,7-dimethylacridine-9,9'-xanthene] (**9**).

Compound 9: To a stirred solution of **10** (20.0 g, 72.4 mmol) in dry THF (300 mL) was added dropwise *n*-butyllithium (2.6 M, 61.4 mL, 159 mmol) at −78 °C under N_2. The mixture was stirred for 1 h at that temperature. After heating up to 0 °C, xanthone (15.6 g, 79.5 mmol) was slowly added to the solution, and the mixture was further stirred for 1 h at 0 °C and for 2 h at room temperature. The reaction was quenched by addition of a large amount of water. Then, the product was extracted with chloroform and dried over anhydrous Na_2SO_4. After filtration and evaporation, the product was used in the next

reaction without further purification.

This crude product was dissolved in chloroform (300 mL), and methanesulfonic acid (7.7 g, 80.1 mmol) was added to the solution at room temperature. The mixture was refluxed for 1 h under air, and then further reacted for 12 h at room temperature. The mixture was concentrated under reduced pressure. The residue was purified by column chromatography on silica gel (eluent: hexane/chloroform = 3:1, v/v) to afford **9** as a white solid (yield = 18.5 g, 68%). ^{1}H NMR (400 MHz, DMSO-d_6): δ 8.99 (s, 1H), 7.18 (dd, *J* = 6.7, 1.6 Hz), 6.95 (t, *J* = 3.8 Hz), 6.9 (d, *J* = 7.8 Hz, 2H), 6.82 (d, *J* = 8.3 Hz, 2H), 6.78 (d, *J* = 8.2 Hz, 2H), 6.31 (s, 2H), 1.95 (s, 6H). MS (MALDI-TOF): *m*/*z* calcd 375.16 [*M*]$^{+}$; found 374.86.

Table S1 The lowest excited singlet (S_1) and triplet (T_1) energies, oscillator strength (*f*), and transition configurations of **1–5** calculated by TD-DFT at the PBE1PBE/6-31G(d).

compound	state	*E* (eV)	*f*	main configuration		ΔE_{ST} (eV)
MFAc-PPM	S_1	2.54	0.0004	H → L	0.699	0.15
(**1**)	T_1	2.39	0	H → L	0.568	
				H−2 → L	0.360	
MXAc-PPM	S_1	2.59	0	H → L	0.699	0.18
(**2**)	T_1	2.41	0	H → L	0.562	
				H−3 → L	0.366	
MFAc-PM	S_1	2.51	0.0003	H → L	0.702	0.13
(**3**)	T_1	2.38	0	H → L	0.590	
				H−3 → L	0.357	
MXAc-PM	S_1	2.54	0.0005	H → L	0.702	0.15
(**4**)	T_1	2.39	0	H → L	0.583	
				H−3 → L	0.372	
Ac-PM	S_1	2.60	0	H → L	0.702	0.18
(**5**)	T_1	2.42	0	H → L	0.573	
				H−1 → L	0.381	

H → L represents the HOMO to LUMO transition. Excitation configurations with the highest contributions are presented, together with the corresponding transition symmetry and nature of the involved orbitals.

Determination of rate constants.

The PL quantum efficiencies of the prompt (Φ_p) and delayed (Φ_d) components were experimentally obtained from the transient PL decay curves (Figure 6). The lifetimes of the prompt (τ_p) and delayed (τ_d) components were determined by fitting the transient PL decay curves. In the presence of intersystem crossing (ISC) and reverse intersystem crossing (RISC) between the S_1 and T_1 states, the rate constants of the prompt (k_p) and delayed (k_d) components can be expressed by the following formulas:

$$k_\mathrm{p} = \frac{1}{\tau_\mathrm{p}} = k_\mathrm{r}^\mathrm{S} + k_\mathrm{nr}^\mathrm{S} + k_\mathrm{ISC} \quad (1)$$

$$k_\mathrm{d} = \frac{1}{\tau_\mathrm{d}} = k_\mathrm{nr}^\mathrm{T} + \left(1 - \frac{k_\mathrm{ISC}}{k_\mathrm{r}^\mathrm{S}+k_\mathrm{nr}^\mathrm{S}+k_\mathrm{ISC}}\right) k_\mathrm{RISC} \quad (2)$$

where k_r^S and k_nr^S are the radiative and non-radiative decay rate constants of the S_1 state, respectively, and k_ISC and k_RISC are the ISC ($S_1 \rightarrow T_1$) and RISC ($T_1 \rightarrow S_1$) rate constants, respectively. k_r^S and k_ISC are assumed to be much faster than k_nr^T and k_RISC. The Φ_p, Φ_d, and Φ_ISC are given by the following formulas:

$$\Phi_\mathrm{p} = \frac{k_\mathrm{r}^\mathrm{S}}{k_\mathrm{r}^\mathrm{S}+k_\mathrm{nr}^\mathrm{S}+k_\mathrm{ISC}} = \frac{k_r^S}{k_p} \quad (3)$$

$$\Phi_\mathrm{d} = \sum_{k=1}^{\infty}(\Phi_\mathrm{ISC}\,\Phi_\mathrm{RISC})^k\,\Phi_\mathrm{p} = \frac{\Phi_\mathrm{ISC}\,\Phi_\mathrm{RISC}}{1-\Phi_\mathrm{ISC}\,\Phi_\mathrm{RISC}} \cdot \Phi_\mathrm{p} \quad (4)$$

$$\Phi_\mathrm{ISC} = \frac{k_\mathrm{ISC}}{k_\mathrm{r}^\mathrm{S}+k_\mathrm{nr}^\mathrm{S}+k_\mathrm{ISC}} = \frac{k_\mathrm{ISC}}{k_p} \quad (5)$$

From Eqs. 1–5, the following equation for k_RISC can be obtained.

$$k_\mathrm{RISC} = \frac{k_\mathrm{p}k_\mathrm{d}}{k_\mathrm{ISC}}\,\frac{\Phi_\mathrm{d}}{\Phi_\mathrm{p}} \quad (6)$$

Since the Φ_p exhibits almost negligible temperature dependence, we assume that $k_\mathrm{nr}^\mathrm{S} \approx 0$ at 300 K.Based on Eqs. 1–6, k_r^S, k_ISC, and k_RISC can be estimated, as listed in **Table S2**.

Table S2 Rate constants and quantum efficiencies of **1–5** in 18 wt%-doped films[a]

emitter	k_r^S $[s^{-1}]$	k_d $[s^{-1}]$	k_{ISC} $[s^{-1}]$	k_{RISC} $[s^{-1}]$	Φ_p [%]	Φ_d [%]	Φ_{ISC} [%]	Φ_{RISC} [%]
**1**	3.4×10^7	2.6×10^4	4.9×10^7	5.0×10^4	41	46	59	78
**2**	4.1×10^7	2.5×10^3	5.0×10^7	2.4×10^4	45	24	55	44
**3**	3.8×10^7	2.2×10^3	3.8×10^7	3.6×10^4	50	41	50	82
**4**	5.5×10^7	1.4×10^3	3.6×10^7	1.8×10^4	60	30	40	75
**5**	5.7×10^7	1.3×10^3	3.4×10^6	1.1×10^4	63	20	37	54

[a]Abbreviations: k_r^S, radiative rate constant ($S_1 \rightarrow S_0$); k_d, delayed-radiative rate constant ($S_1 \rightarrow T_1 \rightarrow S_1 \rightarrow S_0$); k_{ISC}, intersystem-crossing (ISC) rate constant ($S_1 \rightarrow T_1$); k_{RISC}, reverse ISC rate constant ($T_1 \rightarrow S_1$); Φ_p, quantum efficiency for prompt fluorescence component; Φ_d, quantum efficiency for delayed fluorescence component; Φ_{ISC}, ISC quantum efficiency; Φ_{RISC}, RISC quantum efficiency.

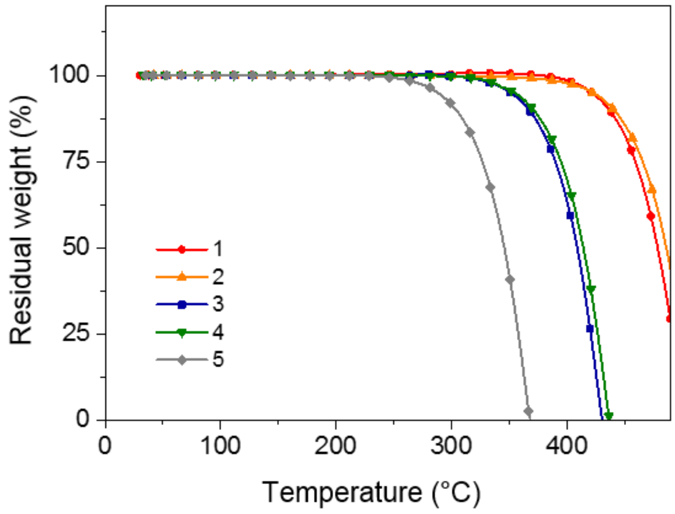

Fig. S1 TGA curves for **1–5** recorded at a heating rate of 10 °C min^{-1} under N_2

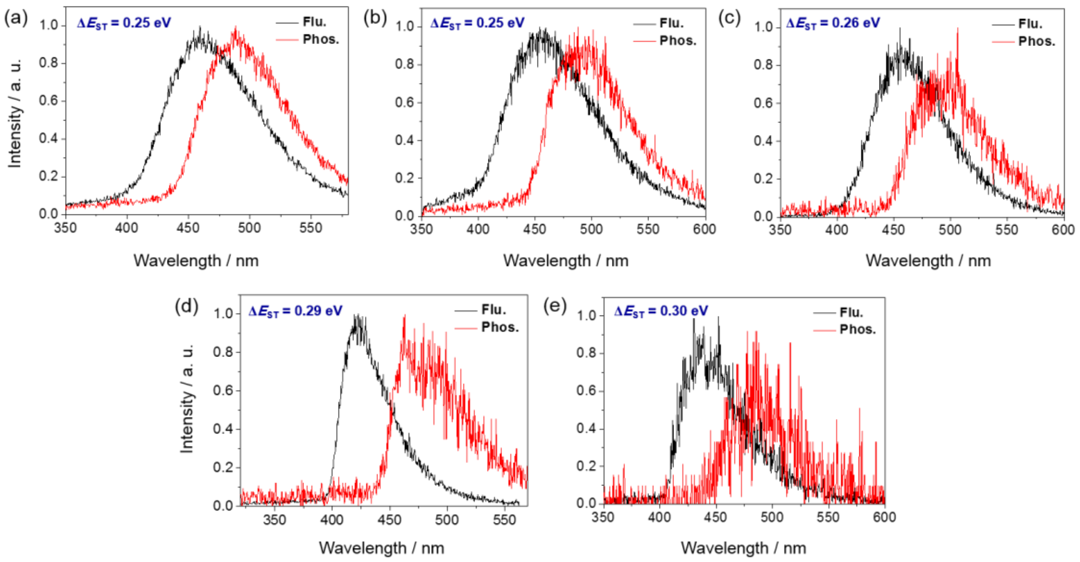

Fig. S2 PL spectra of fluorescence in the time range of 1–100 ns at 300 K (black) and phosphorescence at 5 K in the range of 100–10000 μs (red) for doped thin films of (a) **1**, (b) **2**, (c) **3**, (d) **4**, and (e) **5** in a PPF host matrix. The lowest excited singlet (E_S) and triplet (E_T) energy levels of the TADF emitters were estimated from the high energy onsets of the fluorescence and phosphorescence spectra, respectively.

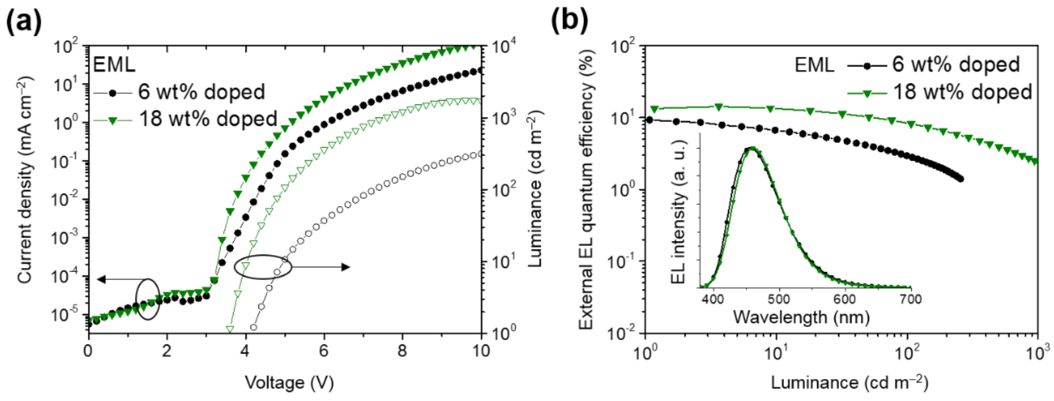

Fig. S3 (a) Current density and luminance versus voltage (*J*–*V*–*L*) characteristics and (b) external EL quantum efficiency versus luminance (η_{ext}–*L*) characteristics of blue TADF-OLEDs based on **4** at a doping concentration of 6 wt% and 18 wt%.

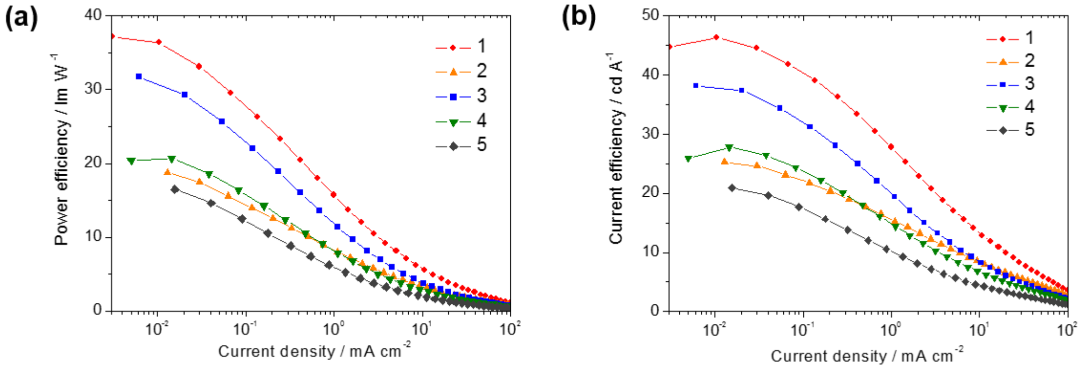

Fig. S4 (a) Power efficiency *versus* current density (η_p–J) plots and (d) Current efficiency versus current density (η_c–J) plots for blue TADF-OLEDs based on **1–5**.

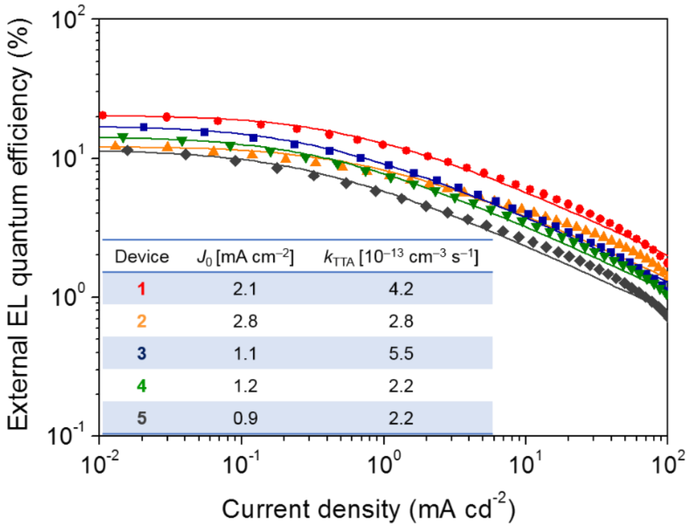

Device	J_0 [mA cm^{-2}]	k_{TTA} [10^{-13} cm^{-3} s^{-1}]
1	2.1	4.2
2	2.8	2.8
3	1.1	5.5
4	1.2	2.2
5	0.9	2.2

Fig. S5 External EL quantum efficiency *verse* current density (η_{ext}–J) plots for blue TADF-OLEDs based on **1–5**. The solid lines represent the simulated η_{ext} by using the TTA model. J_0 is the critical current density at $\eta_{ext} = \eta_0/2$ and k_{TTA} is the TTA rate constant.
